# Notch signaling, hypoxia, and cancer

**DOI:** 10.3389/fonc.2023.1078768

**Published:** 2023-01-31

**Authors:** Mingzhou Guo, Yang Niu, Min Xie, Xiansheng Liu, Xiaochen Li

**Affiliations:** ^1^ Department of Pulmonary and Critical Care Medicine, Tongji Hospital, Tongji Medical College, Huazhong University of Science and Technology, Wuhan, China; ^2^ Key Laboratory of Pulmonary Diseases of National Health Commission, Tongji Hospital, Tongji Medical College, Huazhong University of Sciences and Technology, Wuhan, China

**Keywords:** Notch signaling, hypoxia, hypoxia-induced factors pathway, cancer, therapeutics

## Abstract

Notch signaling is involved in cell fate determination and deregulated in human solid tumors. Hypoxia is an important feature in many solid tumors, which activates hypoxia-induced factors (HIFs) and their downstream targets to promote tumorigenesis and cancer development. Recently, HIFs have been shown to trigger the Notch signaling pathway in a variety of organisms and tissues. In this review, we focus on the pro- and anti-tumorigenic functions of Notch signaling and discuss the crosstalk between Notch signaling and cellular hypoxic response in cancer pathogenesis, including epithelia-mesenchymal transition, angiogenesis, and the maintenance of cancer stem cells. The pharmacological strategies targeting Notch signaling and hypoxia in cancer are also discussed in this review.

## 1 Introduction

The discovery of Notch signaling dates back to the early 1900s when a specific *Drosophila* wing phenotype showed notches on the wings which resulted from the mutations in the Notch receptor. Meanwhile, several other mutations have also been identified, such as Delta and Serrate, which similarly turned out to reside in genes encoding ligands related to the Notch pathway (1). Studies of the Notch signaling have flourished since then and the principal components and process of the signaling transduction cascade were identified. As a juxtacrine signaling, Notch signaling relies on the interaction between receptors and ligands expressed on juxtaposed cells to initiate signaling. The Notch signaling has been extensively characterized as a highly conserved pathway involved in cell proliferation, fate, differentiation, and stem cell maintenance (2). It is universally acknowledged that the normal Notch signaling is vital to most developmental decision-making in animals, and that pathway dysfunction is involved in many conditions, including cancer (3).

The Notch signaling pathway plays a critical role in tumor initiation and progression. Notch can function as an oncogene or a tumor suppressor in different cancers. Hypoxia is a common feature in a majority of malignant tumors. Hypoxia triggers a complex signaling network in tumor cells to alter cell metabolism and regulate angiogenesis, epithelia-mesenchymal transition (EMT), and the maintenance and functions of cancer stem cells (CSCs). Hypoxia-induced factors (HIFs), as global regulators of cellular hypoxia responses, can interact with Notch and directly regulate the Notch signaling pathway. This review systematically summarizes the intersection between Notch signaling and the cellular hypoxic response and highlights the underlying molecular mechanisms involved in the cancer pathogenesis, which contributes to the discovery and development of a combinational strategy targeting Notch and hypoxia in cancer treatment.

## 2 Notch signaling pathway

Notch signaling exerts its effect in a canonical or noncanonical fashion. The specific mechanisms of canonical and non-canonical Notch signaling are described as follows.

### 2.1 Canonical Notch signaling

**Figure 1:**
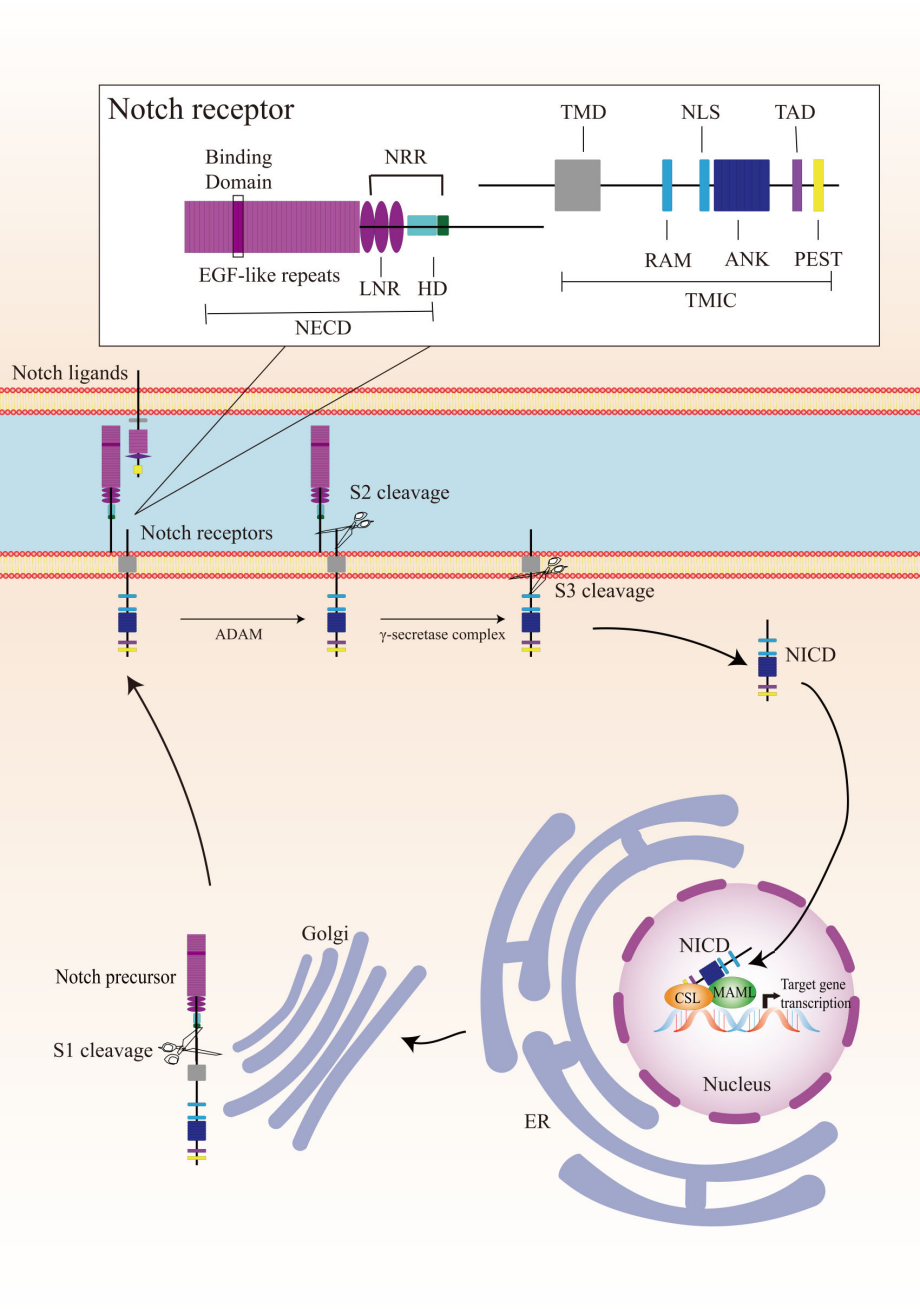
Overview of the Notch signaling pathway. The Notch receptor is produced in the endoplasmic reticulum (ER) and undergoes S1-cleavage in the Golgi compartment. The cleavage results in the formation of a heterodimer receptor, consisting of a Notch extracellular domain (NECD) and a Notch transmembrane and intracellular domain (TMIC), which is then transported to the plasma membrane. Upon interacting with a transmembrane ligand, the Notch receptor undergoes two sequentially cleavage, releases the Notch intracellular domain (NICD), which translocates into the nucleus. In the cell nucleus, NICD forms a ternary complex with the DNA-binding protein CSL and MAML to regulate transcription of downstream genes. A detailed description of the various domains in Notch receptor is presented in the box on the top. Notch receptor consists of a NECD, a transmembrane domain (TMD), and a NICD. NECD consists of epidermal growth factor (EGF) - like repeats domain, and a negative regulatory region (NRR), which including three Lin Notch repeats (LNR) and a heterodimerization (HD) domain. EGF-like repeats 11 and 12 function as specific protein binding domains mediating interaction with ligands. NICD consists of a RBPJ associated molecule (RAM), ankyrin repeats (ANK), a translational active domain (TAD), and a PEST domain.

Canonical Notch signaling is initiated by γ-secretase-mediated cleavage of the Notch receptor, resulting in the release of the active intracellular domain of Notch, which migrates to the nucleus and interacts with CSL (for CBF1, Suppressor of Hairless, Lag1; also known as RBPJ), leading to the activation of downstream target genes (**Figure 1**). In mammals, there are four Notch receptors (Notch 1/2/3/4) and five ligands (Delta-like 1/3/4 or Jagged 1/2). The Notch receptors and ligands are structurally related in some ways. They both contain a large number of epidermal growth factor (EGF)-like repeats in their extracellular domains. Briefly, Notch receptors are produced in the endoplasmic reticulum and synthesized as single precursor proteins, which are then trafficked to the Golgi compartment. In the Golgi compartment, Notch receptor precursors undergo S1 cleavage by a furin-like protease, creating the heterodimeric Notch receptor consisting of a Notch extracellular domain (NECD) and a Notch transmembrane and intracellular domain (TMIC). The part of the extracellular domain of Notch receptor consists of 36 EGF-like repeats and a negative regulatory region. EGF-like repeats 11 and 12 function as specific protein binding domains mediating interaction with ligands (4). The ligand-receptor interaction triggers proteolytic cleavages by an ADAM metalloprotease (S2-cleavage). In this process, ligand will be endocytosed after it binds to Notch receptor. Epsin-dependent ligand endocytosis exerts force on the negative regulatory region exposing the S2 site for cleavage (5). Then, the remainder of the receptor subjected to S3 cleavage by the γ-secretase complex releases the Notch intracellular domain (NICD), which translocates into the nucleus. In the nucleus, NICD interacts with a DNA-binding protein CSL, converting CSL from a transcriptional repressor to an activator. The NICD-CSL interaction is stabilized by Mastermind like transcriptional coactivator (MAML), forming a ternary NICD/MAML/CSL complex to activate the transcription of downstream genes including Hes (hairy-enhancer of split), Hey (Hes related to YRPW), and so on (6, 7). Different ligands could generate diverse Notch activity dynamics in signaling receiving cells, inducing different cell fates *via* activating distinct target gene programs (8).

### 2.2 Non-canonical Notch signaling

Non-canonical Notch signaling is an important arm of Notch signaling. Notch is proved active in cells where the canonical ligands and downstream effectors were defective, indicating that Notch acts in a second way independently (9). Non-canonical Notch signaling can be initiated by a non-canonical ligand *via* CSL-independent manner (10–12).

Notch signaling can be elicited by diverse non-canonical ligands, including ligands structurally similar to canonical ligands, structurally unrelated ligands, and secreted proteins (13, 14). Delta like non-canonical Notch ligand 1 is an integral membrane protein containing tandem EGF-like repeats in its extracellular domain but lacking the DSL domain. It can directly interact with Notch1 and act as an antagonist (14). Another structurally similar non-canonical ligand Delta/Notch-like EGF-related receptor functioned as a trans-ligand to affect glial morphological changes (15). A diverse group of structurally unrelated non-canonical ligands have also been identified as Notch activators. F3/contactin1 and NB3/contactin6 interacted with Notch EGF-like repeat distal to the DSL domain binding site to induce oligodendrocyte differentiation (11, 16). In addition, a number of secreted proteins act as non-canonical ligands of Notch. In vertebrates, CCN3 and MAGP-2 can bind to the extracellular domains of Notch receptor, resulting in its cleavage and activation (17, 18). In *Drosophila*, Scabrous activated transcription of the Notch target gene E(spl)C m3 to regulate eye ommatidia and sensory bristles (19, 20).

In CSL-independent non-canonical Notch signaling, the cleaved NICD interacts with multiple pathways and regulates cell survival. The CSL knockout mice developed breast tumors similar to CSL heterozygous and control mice, indicating that Notch-induced breast tumor development was CSL-independent (21). Interleukin-6 has been identified as a novel Notch target in breast tumor cells. The Notch-mediated interleukin-6 up-regulation required two NF-κB signaling-related proteins and P53 (22). The membrane-tethered NICD inhibited cell apoptosis through interacting with mTOR and Rictor (companion of mTOR) to trigger Akt phosphorylation in activated T cells (23). Notch activated the PI3K-Akt pathway *via* Deltex1 and played oncogenic functions in cervical cancer (24). In addition, Notch1 was demonstrated to directly regulate vascular barrier function through a flow-mediated, non-canonical, transcription-independent signaling mechanism (25, 26).

## 3 Notch signaling pathway in cancer

The mutations in the Notch signaling pathway genes and dysregulated Notch signaling pathways exhibit dual biological functions in tumorigenesis and cancer progression (**Table 1**). Notch1 mutation was first identified in patients with acute T-cell acute lymphoblastic leukemia (T-ALL) and occurs in approximately 50% of T-ALL (27). Oncogenic and gain-­of-­function mutations of Notch genes have been implicated in chronic lymphocytic leukemia (30), splenic marginal zone B-cell lymphoma (31), squamous cell lung carcinoma (44) and salivary adenoid cystic carcinomas (58). Moreover, aberrant activation of Notch signaling has been found in many solid tumors including prostate (59), breast (60), cervical (61), melanoma (62), and lung cancer (63, 64).

**Table 1:** The Oncogenic and tumor suppressive roles of Notch signaling in human cancers.

Tumor Type	Oncogenic or Tumor Suppressive	Mutations
Acute lymphoblastic T-cell leukemia	Oncogenic	Notch1 (27), Notch3 (28), FBXW7 (29)
Chronic lymphocytic leukemia	Oncogenic	Notch1 (30)
Splenic marginal zone lymphoma	Oncogenic	Notch2 (31)
Diffuse large cell B lymphoma	Oncogenic	Notch1 (32), Notch2 (33)
Adenoid cystic carcinoma	Oncogenic	Notch1, Notch2 (34, 35)
Breast cancer	Oncogenic	Notch1, Notch2 (36)
Infantile myofibromatosis	Oncogenic	Notch3 (37)
Glomus tumors	Oncogenic	Notch1, Notch2, Notch3 (38)
Head and neck squamous cell carcinomas	Tumor Suppressive	Notch1 (39, 40)
Small cell lung cancers	Tumor Suppressive	Notch1, Notch2, Notch3, Notch4 (41)
Bladder cancer	Tumor Suppressive	Notch1, Notch2, Notch3, MAML (42, 43)
Cutaneous and lung squamous cell carcinoma	Tumor Suppressive	Notch1, Notch2 (44)
Cholangiocellular carcinoma	Oncogenic	No mutations (45)
Hepatocellular carcinoma	Oncogenic and Tumor Suppressive	No mutations (46–48)
Pancreatic ductal adenocarcinoma	Oncogenic and Tumor Suppressive	No mutations (49, 50)
Melanoma	Oncogenic	No mutations (51, 52)
Prostate cancer	Oncogenic	No mutations (53, 54)
Glioblastoma	Oncogenic	No mutations (55–57)

MAML, Mastermind like transcriptional coactivator.

In addition, Notch signaling can interact with other signaling pathways to promote tumorigenesis and cancer progression (**Table 2**). The Notch signaling contributed to the development of leukemia and breast cancer through interacting with the NF-κB pathway (22, 65, 82). Notch inhibited cervical cancer cell apoptosis *via* the mTOR–Rictor pathway (23).

**Table 2:** The cross-talk between Notch signaling and other pathways in cancers.

Interaction with other pathways	Tumor Type
NF-κB pathway	Leukemic T cells (65), prostate cancer (66), breast cancer (22)
PI3K/Akt pathway	Cervical cancer (24), melanoma (67), breast cancer (68), lung adenocarcinoma (69)
Wnt/β-catenin pathway	Colorectal cancer (70)
HIF pathway	Pancreatic cancer (71), breast cancer (72), glioblastoma (73)
MAPK pathway	Melanoma (67), thyroid papillary cancer (74), breast cancer (75), head and neck squamous cell carcinoma (76)
TGF-β/smad pathway	Breast cancer (77), clear cell renal cell carcinoma (78)
mTOR pathway	Cervical cancer (23)
P53 pathway	Lung adenocarcinoma (79), keratinocyte cancer (80), T-cell lymphoma (81)

In addition to its oncogenic role in human malignancies, Notch also functions as a tumor suppressor (83). Nicolas et al. has demonstrated that Notch1 deficiency in skin resulted in the sustained expression of Gli2 and derepressed β-catenin signaling, causing the development of tumor (84). In addition, Notch was reported to play a suppressive role in B cell ALL (85), human hepatocellular carcinoma (86), small cell lung cancer (41), and neuroendocrine tumors (87). In a word, Notch acts as an oncogene or tumor suppressor in cancer depending on different contexts. To comprehend the full spectrum of Notch effects, efforts were required to identify the specific ligand-receptor interactions, the downstream targets of Notch signaling, and the functions of Notch modifiers (88).

Tumor microenvironment is comprised of a complex network, including stromal cells, immune cells, fibroblasts, blood vessels, and secreted factors (89). The interaction between tumor cells and tumor microenvironment (TME) is interdependent. A normal TME has a potential to suppress tumors. Lim et al. has suggested that tumor-stroma interactions can drive disease progression in squamous cell carcinoma arising in different tissues, indicating that the tumor context defines metastatic progression (90).

Accumulating evidence suggested that Notch signaling plays a role in regulating the immune responses in tumors, which may be associated with the critical role of Notch signaling in hematopoiesis and immune development (88, 91). A single-cell RNA-sequencing analysis has revealed that Jagged1-Notch pathway regulated immune cell homeostasis during minimal residual disease in hematologic neoplasm, which was a potential target to delay tumor recurrence (92). In breast cancer, the Jagged1-Notch pathway regulated tumor-associated macrophage differentiation towards M2 phenotype to induce aromatase inhibitor resistance (93). Activation of the Notch signaling in triple-negative breast cancer resulted in the secretion of pro-inflammatory cytokines and the recruitment of pro-tumoral macrophages to the TME (94). Delta-like 1 (Dll1)-mediated Notch signaling was implicated in the crosstalk between tumor cells and cancer-associated fibroblasts to promote radio-resistance in breast cancer (95). In general, Notch signaling plays a critical role in regulating tumor cells and TME, which may provide new strategies for Notch-targeted cancer therapy.

## 4 Hypoxia in cancer

Oxygen is indispensable for mammals that maintain intracellular ATP levels and serves as an electron acceptor in a large number of biochemical reactions (96). Hypoxia is a major feature of solid tumor and associated with poor prognosis and resistance to therapy (97–99). Under hypoxic condition, tumor cells undergo various biological processes including cell proliferation, migration, apoptosis, and EMT (100). Hypoxia also triggers multiple signaling pathways to regulate advanced but dysfunctional vascularization in TME (101).

The transcriptional factor HIFs are principal regulators and orchestrate cellular adaptive mechanisms in responses to hypoxia. HIFs contain two different subunits: α and β. The α-subunit protein is regulated by cellular oxygen levels, whereas the β subunit is constitutively expressed (102, 103). HIF-α proteins are oxygen-sensitive that contain an oxygen-dependent degradation domain with target prolyl residues, and a C-terminal transactivation domain which contains the target asparaginyl residue. Under normoxic condition, HIF-α subunits are hydroxylated by prolyl hydroxylases. After hydroxylation, the von-Hippel Lindau tumor suppressor gene interacts with HIF-α and tags it for 26s proteasomal degradation (104, 105). Under hypoxic condition, HIF-α hydroxylation is prevented due to the inactivation of prolyl hydroxylases, resulting in the inhibition of ubiquitin-mediated proteasome degradation of HIF-α. HIF-α is stabilized and form the HIF heterodimer, which then enters the nucleus and combines with hypoxia-response elements to activate the downstream genes (106). Moreover, HIF transcriptional activity is modulated by factor inhibiting HIF-1 (FIH-1), which hydroxylates an asparagine residue in the transactivation domain of HIF-α subunits, thereby blocking its transactivation function (107, 108).

There are three known α subunits (HIF-1α, HIF-2α, and HIF-3α) and three β subunits (HIF-1β, HIF-2β, and HIF-3β). HIF-1α is widely expressed in most human tissues, while HIF-2α and HIF-3α are detected in more restricted tissues, such as lung, kidney, and so on (109, 110). In canonical HIF signaling, hypoxia leads to the stabilization of the labile protein HIF-1α or HIF-2α which complexes with HIF-β, forming heterodimers that bind to hypoxia-response elements in target genes (111). HIF-1α and HIF-2α are structurally closely related and share both common and distinct target genes (112). The role of HIF-3α in the regulation of the HIF pathway is not completely understood and mainly regarded as a negative regulator of HIF-1α and HIF-2α (113).

HIFs are overexpressed and significantly associated with poor prognosis in a variety of cancers (114–117). HIFs-regulated genes encode proteins involved in critical aspects of cancer biology, including energy metabolism, cell survival and invasion, angiogenesis, EMT, and so on. Tumor cells tend to turn metabolism from an oxygen-dependent tricarboxylic acid cycle to glycolysis (118). HIF-1 regulates glycolytic enzymes, including hexokinase 2 and phosphofructokinase 1, which involved in tumor initiation and growth (119, 120). A number of growth factors regulated by HIFs played a role in cell survival, such as transforming growth factor-β, insulin-like growth factor 2, endothelin-1, erythropoietin, and epidermal growth factor receptor (100, 121–123). HIFs mediated angiogenesis *via* activating the transcription of multiple angiogenic growth factors, including vascular endothelial growth factor (VEGF), placenta-like growth factor, angiopoietin (124, 125). HIF-1 can directly induces the transcription of ZEB1, TWIST, and TCF3, which promote EMT in cancers (126–128). In a word, HIFs play a key role in cancer initiation and progression.

## 5 Crosstalk between Notch signaling and hypoxia pathway

HIF signaling pathway is the primary regulator in the physiological and pathological response to hypoxia. The Notch signaling pathway plays a critical role in cell fate control, including tumorigenesis and progression. The link between Notch signaling and hypoxia was first described in a transcriptomic analysis, in which the Notch target gene Hes1 was upregulated in hypoxic neuroblastoma cell lines (129). Thereafter, a study of Notch and hypoxia-activated genes in glioblastoma tumor confirmed a combined gene signature of these two pathways and their role in tumor prognosis (130). Gustaffson et al. provided important evidence that hypoxia directly regulated Notch signaling (131). In this study, HIF-1α was recruited to Notch-responsive promoters and interacted with NICD, leading to stabilization of NICD and activation of Notch downstream genes (Hes and Hey). HIF-1α can also be recruited to the Hey-2 promoter in myogenic cell (131). The up-regulation of the Notch ligands (Jagged 2 and Delta-like 4) induced by hypoxia leaded to activation of Notch signaling (132–134). HIF-2α promoted stem phenotype conversion and resistance to Paclitaxel by activating Notch and Wnt pathways in breast cancer cells (72). Besides, HIF-1α was revealed to interact with γ-secretase and upregulate γ-secretase activity to promote cell invasion and metastasis through a novel function independent of transcription factor (135). HIF-1α and HIF-2α synergized with the Notch co-activator MAML1 to potentiate Notch activity in breast cancer cells (136). The indirect regulation of Notch signaling by HIF was reported in lung cancer cells that HIF-mediated miR-1275 up-regulation exerted its tumorigenic effect through co-activating Notch and Wnt/β-catenin signaling pathways (137).

On the other hand, Notch signaling can also regulate hypoxic response. Notch was demonstrated to transcriptionally upregulate the expression of HIF-2α in certain tumor cells *via* a HIF1α-to-HIF2α switch (138). The γ-secretase inhibitor of Notch decreased the mRNA expression of the HIF-1 target PGK-1 (131).

FIH-1 is involved in the crosstalk between hypoxia and Notch signaling pathways. Both HIF-1α and Notch are substrates for the asparagine hydroxylase FIH-1. Two asparagine residues in the NICD ankyrin repeat domain are hydroxylated by FIH-1, leading to inactivation of Notch signaling. FIH-1 binds to NICD more efficiently than HIF-1α, indicating that NICD sequesters FIH-1 away from HIF-1α, which results in an under-hydroxylation on HIF1α (139, 140). This may shed light on another oxygen-dependent interface that modulates HIF signaling.

**Figure 2:**
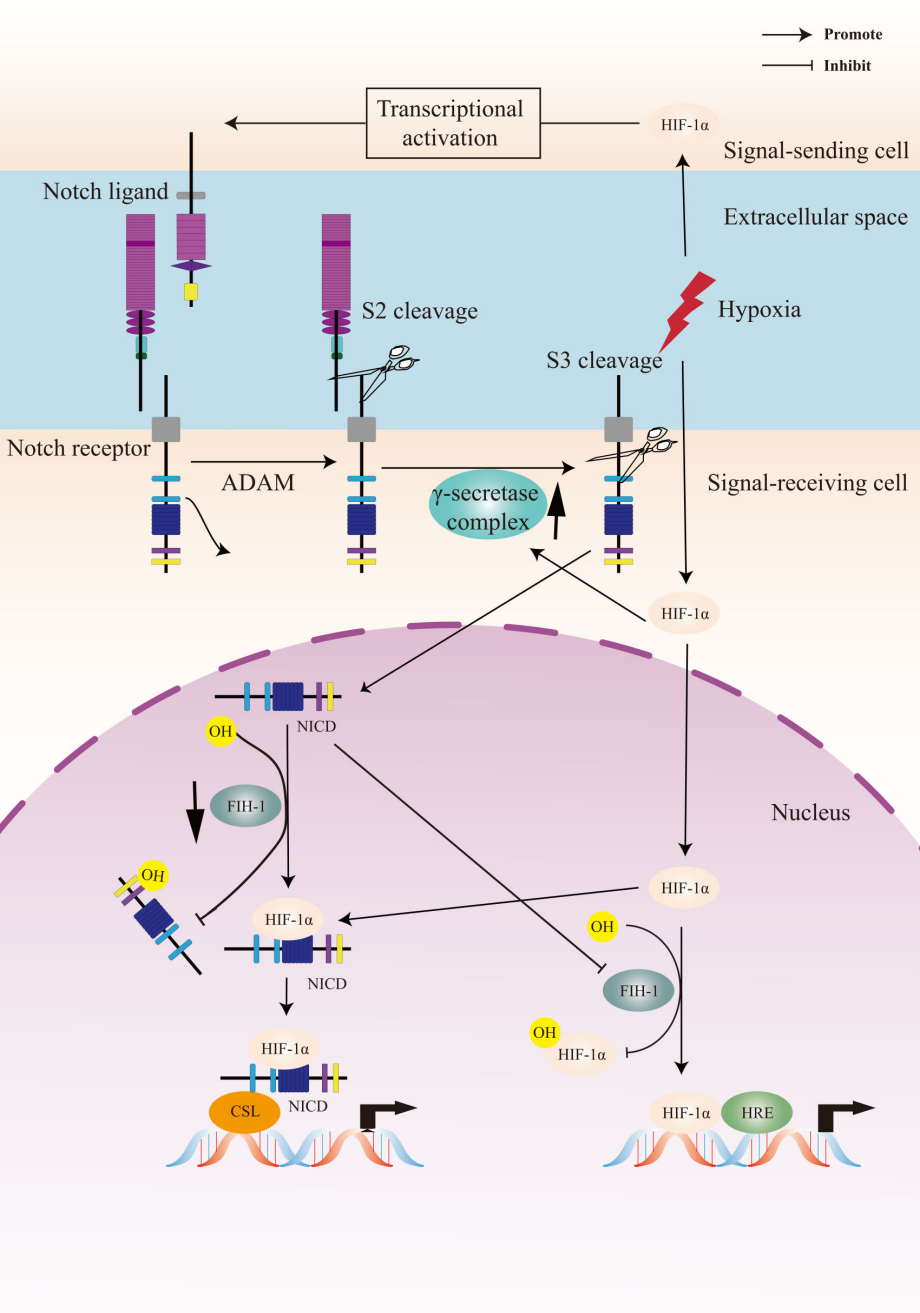
A Crosstalk between Notch signaling and hypoxia pathway. Upon activation of the Notch receptor, the Notch intracellular domain (NICD) accumulates in the cell nucleus and activates target genes. Hypoxia induces the canonical hypoxia response pathway, which involves the activation of hypoxia response element (HRE)-driven target genes. Under hypoxic conditions, hypoxia-induced factors-1α (HIF-1α) potentiates Notch-dependent activation of target genes through interaction with the NICD. Besides, HIF-1α interacts with γ-secretase and upregulated γ-secretase activity. Factor-inhibiting HIF-1 (FIH-1) hydroxylates the asparagine residues of HIF-α and NICD, leading to inactivation of Notch and hypoxia signaling pathways. Hypoxia decreases the activity of FIH-1. In addition, FIH-1 binds NICD more efficiently than HIF-1α. NICD sequesters FIH-1 away from HIF-1α, indirectly resulting in an activation of HRE-driven target genes.

To summarize, the crosstalk between Notch signaling and the cellular hypoxic response is extensive and the underlying molecular mechanism is complex (**Figure 2**). A Notch-hypoxia crosstalk has been involved in a variety of physiological situations and pathological conditions, including vascular diseases and cancers (64, 141).

## 6 Biological processes in cancer regulated by a Notch-hypoxia crosstalk

A functional relationship between hypoxia and Notch signaling pathways has been observed in many types of tumors. Accumulating evidences have revealed that the crosstalk between Notch and the cellular hypoxic response has diverse roles in cancer pathogenesis by regulating several important biological processes, including EMT, angiogenesis, the maintenance of CSCs, and so on.

### 6.1 A Notch-hypoxia crosstalk in cancer EMT

EMT is one of the critical mechanisms of cancer metastasis (142, 143). The hallmark of EMT is the loss of E-cadherin expression through the up-regulation of its repressors (144, 145). E-cadherin repressors are classified into two groups depending on their effects on the E-cadherin promoter. Snail, Zeb, E47, and KLF8 bind to and repress the activity of the E-cadherin promoter (146, 147), whereas several factors such as Twist, Goosecoid, E2.2, and FoxC2 indirectly repress E-cadherin transcription (148).

HIF-1 was reported to upregulate the expression of Twist to promote EMT (149). A number of studies suggested that hypoxia induced EMT *via* activating Notch signaling in tumor cells (136, 150–152). Notch can regulate the expression of Snail-1 *via* two distinct mechanisms in hypoxia. One relied on the transcriptional up-regulation of Snail-1. The other concerned the protein stabilization of Snail-1 *via* the increase of lysyl oxidase which was transcriptionally regulated by HIF-1α and potentiated by Notch (150). Hypoxia-mediated increase in Snail and Slug required Notch pathway in the initiation of EMT in breast cancer cells (136). HIF-1α can also exert a non-transcriptional function in regulating the expression of NICD and E-cadherin in lung cancer cells (153).

### 6.2 A Notch-hypoxia crosstalk in angiogenesis

Tumor growth is fed by nearby blood vessels. Hypoxia occurs as the tumor grows. New blood vessels are essential for continued primary tumor growth. The ability of forming vasculature has been termed angiogenesis. Activation of endothelial cells was a key step of angiogenesis and a number of growth factors upregulated by HIF were involved in the process, such as VEGF (154).

Notch signaling was activated and played an important role in the process of angiogenesis (155). The expression of Notch ligand Dll4 was much higher in the endothelium of tumor blood vessels compared to nearby normal blood vessels, indicating that Notch signaling were implicated in tumor angiogenesis (132, 156, 157). Dll4 was upregulated by VEGF as a negative feedback modulator, which prevented VEGF-induced overexuberant angiogenic sprouting and branching *via* Notch signaling, guaranteeing the formation of a well-differentiated vascular network (158, 159). HIF­1α-induced basic fibroblast growth factor and VEGF were reported to play a synergistic role in the regulation of Dll4 in tumor cells (156). Hypoxia-induced up-regulation of Dll4 and Hey repressed COUP-TFII (known as a regulator of vein identity) in endothelial progenitor cells, which may contribute to tumor angiogenesis (160). Another Notch ligand Jagged 2 was transcriptionally activated by HIF-1α, which triggered Notch signaling and activated Hey1 to promote vascular development and angiogenesis (133).

### 6.3 A Notch-hypoxia crosstalk in the maintenance of CSCs

CSCs represent a discrete subpopulation of cancer cells with stem cell properties, which is responsible for tumor growth. CSCs are self-renewal and can produce more committed progenitor or “transit-amplifying” cells whose progeny differentiate aberrantly to promote the tumorigenesis (161, 162). Stem cell “niches” are considered as particular microenvironments that maintain the combined properties of CSCs self-renewal and multipotency. The Notch signaling is highly conserved and is critical for cell fate decisions and the maintenance of stem cells (163). HIF stabilization in hypoxic tumor cells can promote stem cell properties, including self-renewal and multipotency partly *via* inducing the expression and activity of the Notch signaling pathway (164–167). Hypoxia-induced the 66-kDa isoform of the SHC gene controlled the expression of Notch3 to regulate the stem cell properties (168). In glioblastomas, HIF-1α played an important role in the hypoxia-mediated maintenance of glioma stem cells *via* the interaction with NICD (73). A further study suggested that hypoxia can promote glioma stem cells proliferation and maintain the characteristics of stem cells through activating Notch1 and Oct3/4 (169). In addition, HIF-1α was reported to promote pancreatic cancer cell dedifferentiation into stem-like cell phenotypes by activating Notch signaling, revealing a novel regulatory mechanism (71).

## 7 Strategies for cancer therapy

### 7.1 Therapeutic targets in the Notch signaling pathway

**Figure 3:**
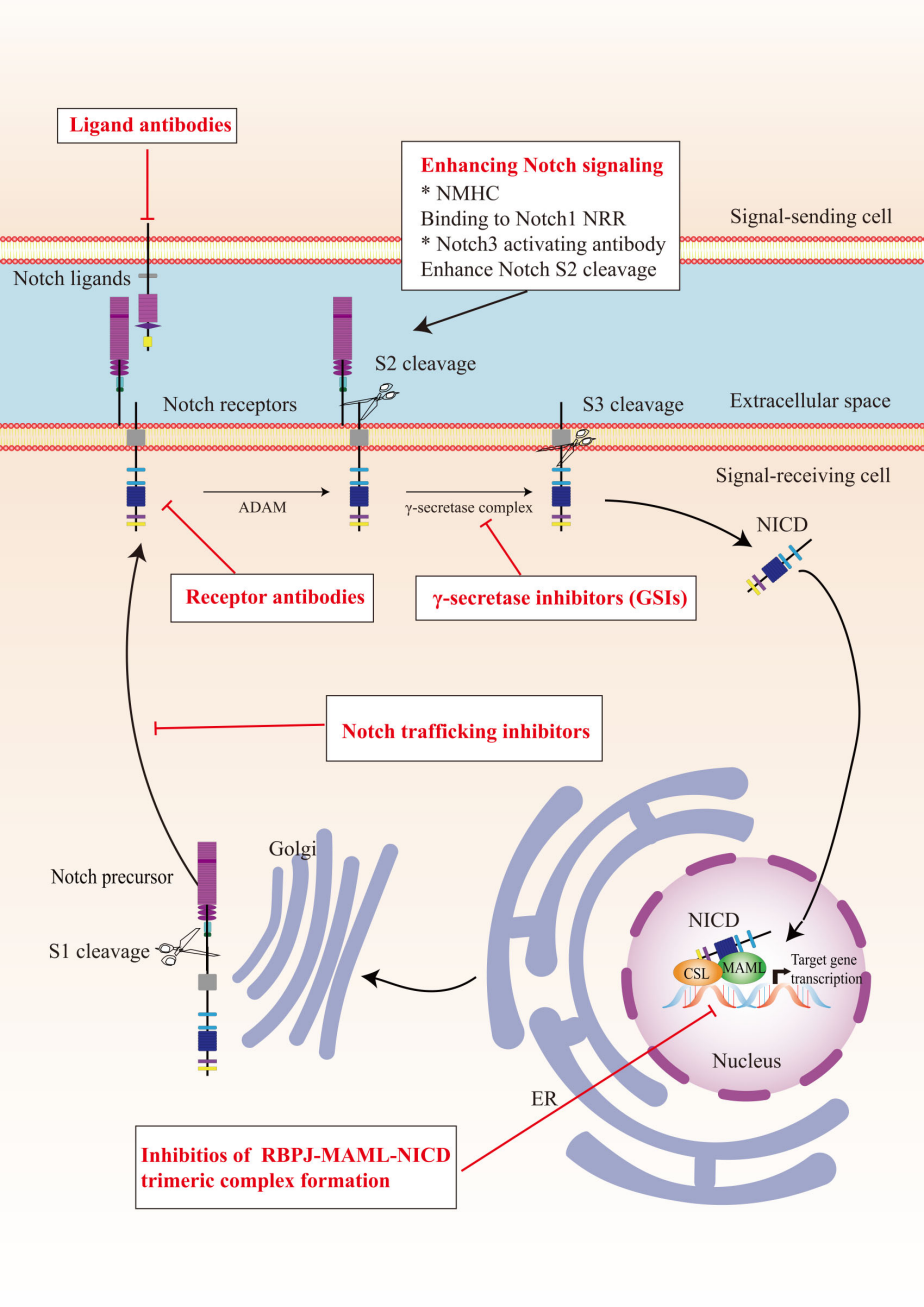
The potential therapeutics targeting Notch signaling pathway. Here are several strategies to modulate Notch signaling pathway: (I) inhibitors of Notch pre-processing, (II) receptor and ligand antibodies blocking ligand-receptor interaction, (III) inhibitors of the trimeric transcriptional complex assembly, (IV) molecules activating Notch signaling. ER, endoplasmic reticulum; NICD, Notch intracellular domain; NRR, negative regulatory region; NMHC, N-methylhemeanthidine chloride; MAML, Mastermind like transcriptional coactivator; GSIs, γ-secretase inhibitors.

In view of the critical role of Notch signaling in tumor pathogenesis, Notch is regarded as a promising therapeutic target. Numerous approaches have been developed to inhibit different steps of Notch signaling pathway for therapy: γ-secretase inhibitors (GSIs), antibodies targeting ligands or receptors, compounds targeting transcription activation, and so on (**Figure 3**). The drugs are listed by therapeutic category in **Table 3**.

**Table 3:** Therapeutic approaches targeting Notch signaling pathway.

Class	Target	Tumor type
GSIs	γ-secretase	T-cell acute lymphoblastic leukemia (170), breast cancer (171), lung adenocarcinoma (172), colorectal cancer (173), prostate cancer (174)
Transcription blocker	CSL/NICD complex	Hematologic cancer (175), breast cancer (176)
Antibodies against Notch receptors	Notch1	T-acute lymphoblastic leukemia (177), adenoid cystic carcinoma (178)
	Notch2/Notch3	Untreated metastatic pancreatic cancer (179), small cell lung cancer (180), and other solid tumors (181)
	Notch3	Advanced breast cancer and other solid tumors (182)
Antibodies against Notch ligands	Jagged-1	Breast cancer (183), and other malignant tumors (184)
	Delta-like ligand 3	Small cell lung cancer (185)
	Delta-like ligand 4	Ovarian cancer (186), Metastatic non-squamous non-small cell lung cancer (187), and other advanced solid tumors (188)
Enhance Notch signaling activation	Notch negative regulatory region	Acute myeloid leukemia (189)
Therapeutic non-coding RNAs	MiRNAs	Prostate cancer (190), breast cancer (191), ovarian cancer (192), pancreatic cancer (193)
	LncRNAs	Ovarian cancer (194), nasopharyngeal carcinoma (195)

GSIs=γ-secretase inhibitors; miRNAs=microRNAs; lncRNAs=long non-coding RNAs.

GSIs were the first and most extensively studied small-molecule Notch inhibitors. Initially, GSIs were developed for treating Alzheimer’s disease because γ-secretase catalyzed the production of the β-amyloid peptide from amyloid precursor protein (196). The use of GSIs for cancer treatment is based on inhibiting the cleavage of γ-secretase which mediates S3 cleavage to generate NICD, resulting in blocking Notch signaling. However, studies have shown that systemic inhibition of Notch signaling by GSIs results in “on-target” gastrointestinal toxicity because of the accumulation of secretory goblet cells in the intestine. The above observation can be explained by alterations in the differentiation of intestinal stem cells following the dual inhibition of Notch1 and Notch2 (197). Co-administration of glucocorticoid may alleviate the toxicity through inducing transcriptional up-regulation of cyclin D2 and protecting mice from developing the GSIs-induced intestinal goblet cell metaplasia in a preclinical mouse model of T-ALL (198).

Considering the inherent mechanism-based toxicity caused by pan-Notch inhibitor GSIs, novel inhibitors that selectively target individual Notch ligands and receptors have been developed. Selective blocking of Notch1 signaling inhibited cancer cell growth and deregulation of angiogenesis (199). The antibodies against Notch receptors are divided into two classes, one directed against the EGF-like repeat region and the other directed against the Notch negative regulatory region (200). Several potent and selective inhibitors against Notch1, Notch2, and Notch3 have been developed (199, 201, 202). However, there is a lack of inhibitor against Notch 4. The antibodies that selectively target the canonical ligands have also been investigated, such as Jagged antagonism (203).

In the past decades, several molecules targeting Notch trafficking and processing have been developed. The dihydropyridine FLI-06 as the first small molecular chemical compound functioned at an early stage in secretory traffic through disrupting the Golgi apparatus and inhibiting general secretion before exiting from the endoplasmic reticulum (204). FLI-06 was also demonstrated to block Notch activation and decrease the self-renewal ability of tongue CSCs (205). In addition, direct inhibition of the CSL/NICD complex has been reported to treat cancers. SAHM1, as a high-affinity binding of the hydrocarbon-stapled peptide, could prevent the assembly of the active transcriptional complex, resulting in genome-wide suppression of Notch-activated genes for the treatment of leukemia (206). There are other small molecules inhibiting the transcriptional activation complex, which have been investigated, such as IMR-1, CB-103, and RIN1 (175, 176, 207). However, given that loss of CSL derepressed target gene promoter and promoted tumorigenesis, targeting CSL may bring potential problems (208).

As mentioned above, Notch can act as a tumor suppressor in specific contexts, thus enhancing Notch signaling activation is a potential therapeutic strategy for cancer. A study demonstrated that N-methylhemeanthidine chloride, a novel Amaryllidaceae alkaloid, activated the Notch signaling *via* docking in the hydrophobic cavity within the Notch1 negative regulatory region and promoting Notch1 proteolytic cleavage (189). A monoclonal antibody was reported to enhance Notch3 cleavage and mimic the effects of ligand-induced Notch activation *via* binding to overlapping epitopes within negative regulatory region (202).

Accumulating evidence demonstrated that the non-coding RNAs’ (ncRNAs) played a critical role in cancer therapy. NcRNAs are a class of RNAs including microRNAs (miRNAs) and long ncRNAs (lncRNAs) and other short ncRNAs. miRNAs and lncRNAs regulated cell fate determination *via* various signaling pathways (209). miRNA-34 was reported to suppress Notch1 expression, inducing ovarian cancer cell death (210). In contrast, miRNA-223 as an oncogene activated Notch signaling to induce tumor cell proliferation in colorectal cancer (211). The versatility is one of the advantages of miRNA therapeutics, which can suppress or mimic the activity of a miRNA. However, the delivery of miRNA remains an important challenge. LncRNAs mostly act as oncogenes in cancers. LncRNAs can interact with Notch or act as competing endogenous RNAs for miRNAs to indirectly induce Notch signaling in various cancers (212–214). Besides, other therapeutics targeting Notch are currently under investigation, such as natural products, virotherapy, and so on.

### 7.2 Hypoxia targeting strategies

Considering the critical role of hypoxia in tumor initiation, progression and therapy resistance, a growing number of preclinical and clinical cancer studies targeting hypoxia have been performed. In general, the strategies can be classified into hypoxia activated prodrugs (HAPs) and pharmacological inhibitors of the HIF signaling pathway.

#### 7.2.1 Hypoxia activated prodrugs

HAPs are bioreductive drugs which are reduced by specific reductases under hypoxic conditions and release cytotoxins to kill cells (215). Five different chemical entities have the potential to target hypoxia based on their enzymatical reductive reaction under hypoxic conditions (216), including nitro groups, quinones, aromatic N-oxides, aliphatic N-oxides and transition metals. To date, several HAPs have been developed, including EO9 (apaziquone), RH1, SR 4233 (tirapazamine), SN30000, AQ4N (banoxantrone), PR-104, and TH-302 (evofosfamide) (**Table 4**). The effects of HAPs are different depending on the degree of hypoxia and the activity of reductase enzymes. The selection of the appropriate agents in different patients is dependent on the clinical context and requires predictive biomarkers (225).

**Table 4:** Hypoxia-activated prodrugs in clinical development.

Class	Prodrug	Current status	Tumor type
Quinone	E09 (Apaziquone)	III	Bladder cancer (217)
	RH1	I	Solid tumors (218)
Aromatic N-oxide	SR 4233 (Tirapazamine)	III	Non-small-cell lung cancer (219)
	SN30000	Preclinical	Triple-negative breast cancer (220)
Aliphatic N-oxide	AQ4N (Banoxantrone)	I	Solid tumors (221, 222)
Nitro	PR-104	II	Acute myeloid leukemia/lymphoblastic leukemia (223)
	TH-302 (Evofosfamide)	III	Soft-tissue sarcomas (224)

#### 7.2.2 Inhibitors of HIF signaling

HIF signaling is an attractive target for cancer treatment. Several inhibitors have been developed to directly bind to HIF-1α or HIF-2α, resulting in inhibition of their heterodimerization with HIF-β, such as acriflavine (226), PT2385 (227) and PT2399 (228). Heat shock protein 90 (Hsp90) can bind to HIF-1α and block the VHL-dependent proteasomal degradation of HIF-1α. A number of Hsp90 inhibitors have been developed during the past two decades. Hsp90 was identified as the biological target of the ansamycin class of natural products and derivatives, which has been extensively studied in cancer treatment (229). Hsp90 inhibitors apigenin and radicicol reduced hypoxia-induced VEGF expression to decrease angiogenesis (230, 231). Hsp90 can also modulate the conformation of the HIF-1 heterodimer, increasing its interaction with hypoxia-responsive elements, inducing HIF-1 transcriptional activity (231). Hsp90 can be regulated by posttranslational modifications, including acetylation. The process of histone acetylation is regulated by opposing activities of histone acetyltransferases and histone deacetylases (HDACs). HDAC6 functions as an Hsp90 deacetylase (232). HDAC inhibitor vorinostat was developed to inhibit HIF-1 transcriptional activity *via* direct Hsp90 acetylation, decreasing Hsp90-HIF-1 affinity and the interaction between HIF and hypoxia-responsive elements (233). Chetomin, a small molecule blocking the transcriptional co-activation of HIF-1 pathway, was evaluated as a promising candidate treatment for several types of cancers (234). Paradoxically, the stabilization of HIF-1α through inhibition of prolyl hydroxylase domain-containing protein 2 has antitumor effects in certain context. The loss of EGLN1 which encodes prolyl hydroxylase domain-containing protein 2 inhibited the proliferation of clear cell ovarian cancer cells (235). In general, anti-HIF agents are classified by different molecular mechanisms, including inhibition of HIF protein synthesis, degradation, and transcriptional activity. A detailed review of experimental chemical compounds and approved drugs directly targeting HIF pathway are presented in **Table 5**.

**Table 5:** Inhibitors directly targeting the HIF pathway in cancers.

Mechanism of inhibition	Compound/drug name	Current status	Tumor type
Inhibit HIF-1α mRNA expression	EZN-2698	I	Advanced malignancies (236)
Inhibit HIF-1α protein expression	Digoxin	II	Biochemically relapsed prostate cancer (237)
	2-methoxyestradiol	II	Multiple types of cancer (238)
	PX-478	I	Advanced solid tumors and lymphomas (239)
Increased HIF-1α degradation	YC-1	Preclinical	Several solid tumors (240)
	PX-12	II	Previously treated advanced pancreatic cancer (241)
	LW6	Preclinical	Colon cancer (242)
Inhibit HIF heterodimerization	Acriflavin	Preclinical	Prostate cancer (226)
	PT-2385	I	Advanced clear cell renal cell carcinoma (243)
	PT-2399	Preclinical	pVHL-defective clear cell renal cell carcinoma (244)
Inhibit HIF-1/DNA binding	Echinomycin	II	Several advanced cancers (245–249)
Inhibit HIF-1 transcriptional activity	Chetomin	Preclinical	Multiple myeloma (250)
	Bortezomib	FDA approved	Multiple myeloma and several solid tumors (251)
	Vorinostat	II	Metastatic urothelial cancer (252)

Targeting HIF signaling can be performed *via* interfering with other signaling pathways. PI3K/AKT/mTOR and MAPK/ERK pathways can increase HIF-1α synthesis in a cell type-specific manner (253). PI3K inhibitors LY294002 and wortmannin have been recognized as the synthesis inhibition of HIF-1α protein in the prostate carcinoma-derived cell lines PC-3 and DU145 (254). Temsirolimus, everolimus, and sirolimus as mTOR inhibitors are currently in clinical development for the treatment of solid tumors (255). The phase III clinical trials for temsirolimus and everolimus have been completed and showed a significant gain in survival for patients of metastatic renal cell carcinoma (256).

### 7.3 Combination therapy

Combination therapy is an important trend in the development of anticancer agents, and targeting hypoxia is critical in the new strategy (225). The anti-hypoxia agents were combined with immune checkpoint inhibitors to enhance the effect of immune checkpoint inhibitors in cancer treatment, which was based on hypoxia-induced expression and activity of immune checkpoints and immune checkpoint ligands on immune-cells and tumor cells (257). A phase II clinical trial of pembrolizumab and HDAC inhibitor vorinostat demonstrated the combination was active for patients with recurrent/metastatic squamous cell carcinomas of the head and neck, and salivary gland cancer (258). In a neuroblastoma xenograft model, the combination of anti-angiogenic drug sunitinib with hypoxia-activated prodrug evofosfamide was demonstrated to improve survival of mice (259).

Hypoxia and cellular interaction between tumor and non-tumor cells are two important TME. There are strong links between these two themes, and hypoxia contributes to TME to adversely affect therapeutic outcomes. Notch signaling plays an important role in regulating the crosstalk between the different compartments of the TME. Therefore, a combination of targeting Notch and hypoxia implies a potential treatment strategy of cancer to alter TME. In addition, hypoxia and Notch signaling have been shown to form a complex web of interaction in cancer, providing new insights into the combination therapeutics. Notch is a key regulator of tumor angiogenesis (260). The anti-angiogenesis drugs aggravated tumor hypoxia (261), indicating that targeting Notch may induce hypoxia. While, hypoxia activated Notch signaling pathway and may reduce the effect of Notch signaling inhibitors. Therefore, the combination of anti-hypoxia and Notch-targeted agents may present a new strategy for addressing the adverse effect of hypoxia.

## 8 Conclusion

The Notch signaling, as an evolutionarily conserved pathway, is usually activated and extensively involved in tumor initiation and progression. Notch signaling plays a critical role in the interaction between the tumor cells and the surrounding TME, acting as an oncogene or a tumor suppressor. Hypoxia is recognized as a hallmark of TME and the HIF pathway is a master regulator of the cellular hypoxic response. The interaction of Notch and HIF pathways played a key role in multiple biological processes in hypoxic tumor, including EMT, angiogenesis, and the maintenance of CSCs. A broad spectrum of anti-hypoxia agents and Notch signaling inhibitors have been developed during the past decades. The combination therapy has been an important trend of cancer treatment. Considering the complex web of hypoxia and Notch signaling, the combination of them implies a potential treatment strategy of cancer.

## Author contributions

XCL and MG conceived the study. MG drafted the manuscript. XCL revised the manuscript critically for important intellectual content. YN, MX, and XSL provided important comments on the manuscript. All authors approved the final version of the manuscript. The corresponding author attests that all listed authors meet authorship criteria and that no others meeting the criteria have been omitted. All authors contributed to the article and approved the submitted version. The reviewer BZ declared a shared affiliation with the authors to the handling editor at the time of review.
